# Epidemiological, clinical, and pathological characteristics of invasive breast cancer in Bedouin and Jewish women in southern Israel: a retrospective comparative study

**DOI:** 10.1186/s12885-024-12051-w

**Published:** 2024-03-06

**Authors:** Itamar Ben Shitrit, Ao Wang, Karny Ilan, Ravit Agassi, Sofyan Abu Freih, Julie Vaynshtein

**Affiliations:** 1 Faculty of Health Sciences, Soroka University Medical Center, Ben-Gurion University of the Negev, Beer- Sheva, Israel; 2 Clinical Research Center, Faculty of Health Sciences, Soroka University Medical Center, Ben- Gurion University of the Negev, Beer-Sheva, Israel

**Keywords:** Breast neoplasm, Bedouin, Mortality, Prevention, Low income

## Abstract

**Background:**

Invasive breast cancer (IBC) is a leading cause of cancer-related death among women in Israel, regardless of ethnicity. This study compared IBC epidemiological, clinical, and pathological characteristics in Bedouin and Jewish patients in southern Israel.

**Methods:**

Medical records of 1514 Jewish and 191 Bedouin women with IBC treated at Soroka University Medical Center between 2014 and 2021 were analyzed retrospectively. Baseline measures and tumor characteristics were compared between groups. Overall survival (OS) and disease-free survival (DFS) were analyzed using log-rank test. Multivariate analysis was performed using the Cox proportional hazard model.

**Results:**

Bedouin patients exhibited a significantly younger age at diagnosis (median 48 vs. 62 years, *p* < 0.001), larger tumor size (median 2.5 vs. 2.13 cm, *p* < 0.001), and higher metastasis rate (18.8% vs. 12.7%, *p* = 0.03) compared to Jewish patients. In early-stage (non-metastatic) disease, Jewish and Bedouin patients had comparable overall survival (OS) rates (127 vs. 126 months, *p* = 0.2), consistent across stages 1 to 3. However, among patients with metastatic disease, Bedouins exhibited significantly longer OS (76.6 vs. 37.8 months, *p* = 0.006). Disease-free survival (DFS) showed no ethnic differences (not reached vs. 122 months, *p* = 0.31). There were no significant differences in OS between Bedouin and Jewish patients undergoing various treatment modalities for early-stage disease: surgery, adjuvant radiotherapy, chemotherapy, and systemic neoadjuvant therapy.

**Conclusion:**

Breast cancer among Bedouin women in southern Israel manifests at a younger age, with larger tumors and more advanced stages than in Jewish women. However, recent data indicate no differences in OS and DFS between the ethnic groups despite past disparities in prognosis.

**Supplementary Information:**

The online version contains supplementary material available at 10.1186/s12885-024-12051-w.

## Introduction

Invasive breast cancer (IBC) is the most frequently diagnosed cancer in women in North America, Australia, Northern and Western Europe, and Israel. Moreover, it is second among the causes of cancer-related deaths in women regardless of ethnic origin [1–3]. In Israel, age-adjusted IBC incidence rates vary among different ethnic groups and are known to be highest among Jewish women of European origin, lower among Jewish women of North African and Asian origin, and lowest among Arab women [4].

The Bedouins are part of the Israeli-Arab population and reside in the Negev, a desert in southern Israel. The Bedouin population accounts for approximately 22% of the 1.3 million population of the Negev, while most Israeli Arabs living in the Negev are Bedouins. They accounted for approximately 4% of the population of Israel in 2021 [5].

IBC accounted for about a third of all invasive tumors in women in Israel according to 2018 data:33% among Jewish and others and 35% among Arab women, with a total of 4,235 Jewish women (90% of patients) and 507 Arab women (10% of patients) diagnosed that year. The population distribution in 2018 was 79% Jewish and other and 21% Arab; however, the age-adjusted incidence rates per 100,000 of IBC were 88.7 in Jewish and others and 69.5 in Arab women. Moreover, observation of the incidence of IBC in Israeli Arabs during 1996–2017 showed an annual increase of 1.7% compared to a decrease of 0.7% in Jewish women each year [6]. A study published in 2014 examined the epidemiology of IBC in Bedouin and Jewish women. The study found that Bedouin women have a worse prognosis compared with Jewish women [1].

Bedouin women in Israel face many barriers to healthcare access, including socio-cultural factors such as gender inequality and marginalization within their community and practical barriers such as a lack of transportation and difficulty communicating with healthcare providers [7, 8]; however, recent studies indicate that the accessibility of healthcare for Bedouin women has improved due to linguistic adaptation of the healthcare system and the increased availability of early cancer diagnosis and treatment in Bedouin settlements and adjacent regions [9, 10]. Moreover, recent publications have shown that the Bedouin population has experienced a marked shift from a semi-nomadic, traditional lifestyle to a more sedentary and urbanized one [11]. Nevertheless, tribal endogamy and intra-familial consanguinity are strongly favored among Bedouins [12, 13]. Therefore, numerous hereditary disorders are common in the Bedouin population of the Negev [8], making the Bedouin population highly interesting for studying IBC.

The present study examines the epidemiology of IBC among Jewish and Bedouin women. Since prior studies assessed similar cancer characteristics among the Jewish and Bedouin population [1, 10], we aimed to assess the most current data available on the epidemiology of IBC among Jewish and Bedouin women treated at Soroka University Medical Center (SUMC). We hypothesized that due to the significant investments of healthcare funds in the southern region of Israel [14, 15] and the transition of the Bedouin population to a more urbanized lifestyle [16], the prognosis of IBC among Bedouin women would be better than described in the past [1].

## Methods

The data in this study were retrospectively collected from Soroka University Medical Center’s (SUMC) electronic medical record system. The inclusion criteria covered Jewish and Bedouin-Arab female patients diagnosed with breast cancer and treated between 1st January 2014 and 31st December 2021 in SUMC, with the exclusion of non-Bedouin Arabs and male patients. The data collection was performed by manual surveillance of the patient’s computerized files. Data was gathered on patient demographics, baseline tumor characteristics, treatment modality, and survival outcome. Survival analysis included follow-up duration, disease-free survival, and survival time. Patients without recorded disease recurrence or death information were excluded from survival analyses. The extent of the disease was reported according to the American Joint Committee on Cancer (AJCC) [17]. This study was approved by the SUMC institutional Helsinki board (approval number: 0008–22 SOR). The research was performed in accordance with the Declaration of Helsinki and all methods were carried out in accordance with relevant guidelines and regulations.

### Statistical analysis

All statistical analysis from this study was done with RStudio 4.3.1+. Descriptive statistics presented demographic and clinical parameters. Continuous variables such as age and tumor size were summarized using mean and standard deviation. Categorical variables were summarized using percentages. Wilcox rank test was used to compare continuous variables (i.e., age and tumor size) between groups. Fisher’s exact test was used to compare the frequency of categorical variables (i.e., recurrence and death rate) between groups. Overall survival (OS) was defined as the time from diagnosis to the time of death from any cause. The log-rank test was used to compare the Kaplan-Meier OS and disease-free survival (DFS) curves between groups. Statistically significant variables in the univariate analyses were included along with ethnicity in the multivariate Cox’s analyses. All statistical tests were two-sided, and statistical significance was defined at a level p less than 0.05.

## Results

A total of 1705 patients were retrospectively analyzed in this study, including 191 Bedouin patients and 1514 Jewish patients. Table 1 summarizes the baseline demographic and tumor characteristics between these two groups. Bedouin patients were significantly younger at the age of diagnosis compared to Jewish patients (49.6 vs. 60.8 years, *p* < 0.001, Figure S1A), a trend that was also evident while comparing those diagnosed with metastatic disease (47 vs. 61 years, *p* < 0.001, Table S1). Bedouin patients also had a larger tumor size (2.5 vs. 2.13 cm, *p* < 0.001, Figure S1B), more lymph node involvement (nodal stage > 1 51.9% vs. 41%, *p* < 0.035), and more distant metastasis (18.8% vs. 12.7%, *p* = 0.03). Bedouin patients showed a significantly higher prevalence of progesterone-receptor (PR)-positive tumors than Jewish patients (77% vs. 69.9%, *p* = 0.04), with no ethnic differences in estrogen-receptor (ER) positivity rates.

**Table 1:** Baseline patient demographics and tumor characteristics. SD, standard deviation; IDC, intraductal carcinoma; ILC, intralobular carcinoma; ER, estrogen receptor; PR, progesterone receptor; Her 2, human epidermal growth factor receptor 2

Patient and Tumor Characteristics	All *N* = 1705	Jewish *N* = 1514	Bedouin *N* = 191	p value
Age, yrs, mean (SD)	59.5 (14.6)	60.8 (14.3)	49.6 (13.8)	**< 0.001**
Tumor type, n (%)				0.26
IDC ILC Others	1489 (87.3%) 149 (8.8%) 67 (3.9%)	1322 (87.3%) 136 (9%) 56 (3.7%)	167 (87.4%) 13 (6.8%) 11 (5.8%)	
ER positive, n (%)	1375 (80.6%)	1221 (80.6%)	154 (80.6%)	1
PR positive, n (%)	1205 (70.7%)	1058 (69.9%)	147 (77%)	**0.04**
HER 2 positive, n (%)	367 (21.5%)	317 (20.9%)	50 (26.2%)	0.11
Triple Negative	175 (10.3%)	161 (10.6%)	14 (7.3%)	0.17
Tumor Stage, n (%)				**0.02**
T1 T2 T3 T4	865 (52.3%) 486 (29.4%) 74 (4.5%) 228 (13.8%)	769 (52%) 433 (30%) 72 (6%) 193 (12%)	96 (51%) 53 (29%) 2 (1%) 35 (19%)	
Tumor Size (cm)	2.17 (1.51)	2.13 (1.49)	2.50 (1.60)	**< 0.001**
Nodal Stage, n (%)				**0.035**
0 1 2 3	921 (57.8%) 503 (31.6%) 121 (7.6%) 49 (3.1%)	836 (60%) 435 (30.7%) 105 (7.4%) 41 (2.9%)	85 (48%) 68 (38.4%) 16 (9%) 8 (4.5%)	
Metastasis	229 (13.4%)	193 (12.7%)	36 (18.8%)	**0.03**

Table 2 summarizes treatment modality and prognostic outcomes for patients with early-stage (non-metastatic) disease. The median follow-up period was 53.2 months for the entire cohort, with similar medians between the Bedouin (53 months) and Jewish (57.2 months) groups (*p* = 0.15). Bedouin patients had a higher rate of receiving adjuvant chemotherapy and systemic neoadjuvant therapy than Jewish patients (57.4% vs. 39.1%, *p* < 0.001; 45.2% vs. 23.3%, *p* < 0.001, respectively). However, there was no difference in the proportion that underwent surgery, adjuvant radiotherapy, or adjuvant hormonal therapy and no difference in cancer recurrence rates.

**Table 2:** Treatment modality and prognostic outcome between Jewish and Bedouin patients for early-stage disease. SD, standard deviation

	All *N* = 1476	Jewish *N* = 1302	Bedouin *N* = 155	p value
Median follow-up, months (SD)	53.2 (33.5)	57.2 (13.1)	53 (14.3)	0.152
Surgery, n (%)	1368	1217 (93.5%)	151 (97.4%)	0.051
Chemo, n (%)	595	506 (39.1%)	89 (57.4%)	**< 0.001**
Systemic Neoadjuvant, n (%)	371	301 (23.3%)	70 (45.2%)	**< 0.001**
Adjuvant Radiotherapy, n (%)	1098	965 (74.6%)	133 (85.8%)	**0.001**
Hormonal, n (%)	1098	976 (75.7%)	122 (78.7%)	0.428
Recurrence, n (%)	248	216 (16.6%)	32(20.6%)	0.214

**Fig. 1:**
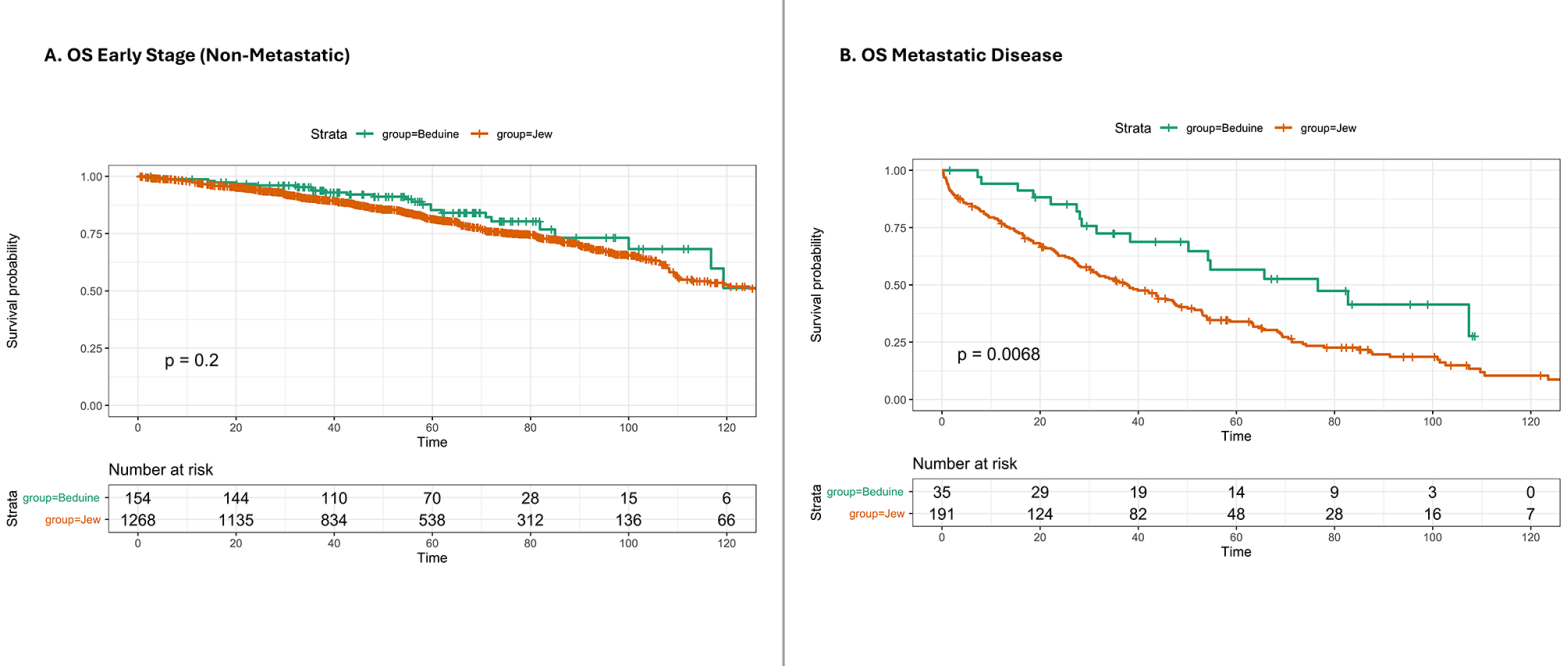
Kaplan-Meier curve of OS in Bedouin and Jewish patients. Panel **A** shows OS rates for early stage (non-metastatic) disease and Panel **B** for metastatic disease. P value is calculated using log-rank test. The survival curve is censored at 120 months. shown. OS, overall survival

**Fig. 2:**
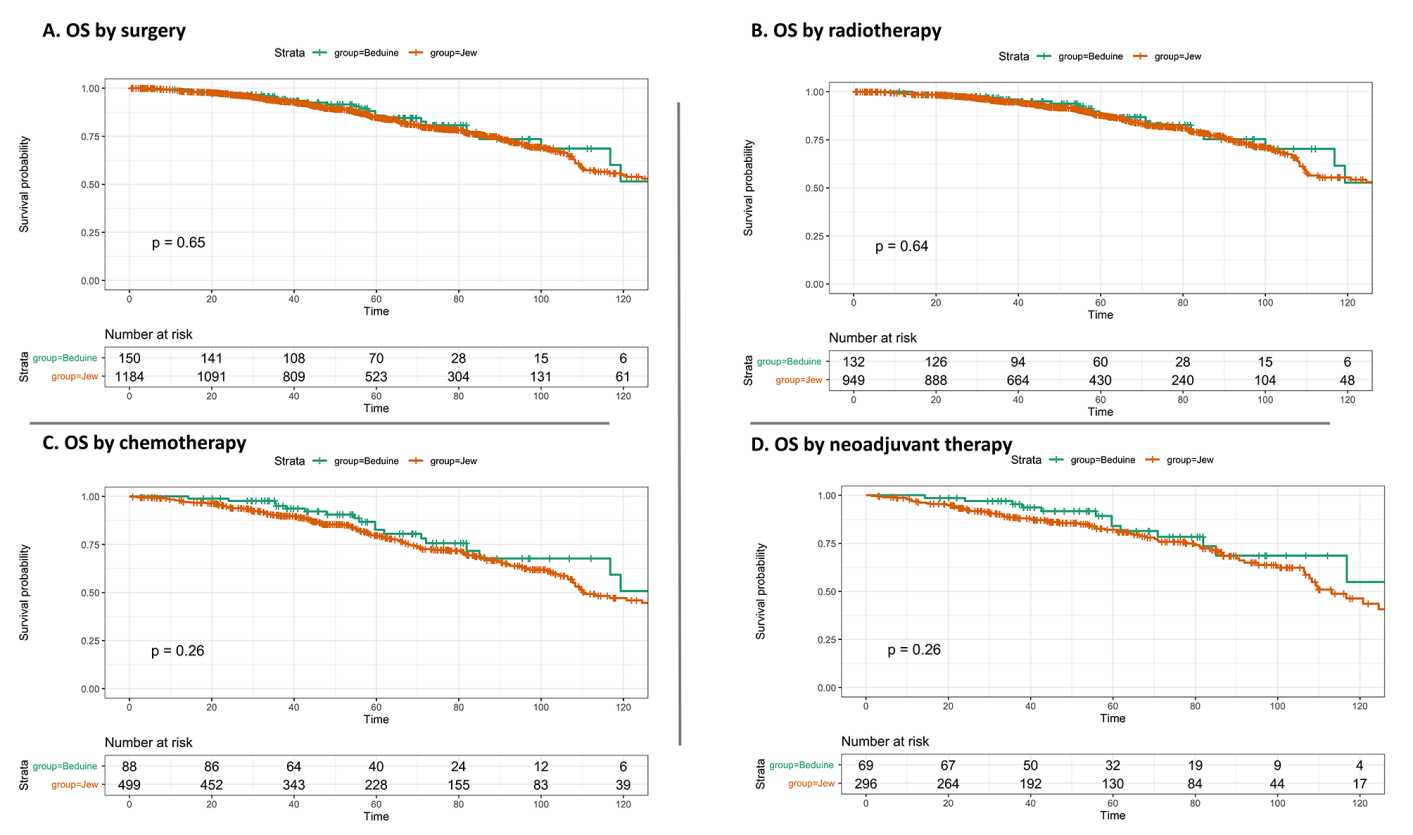
Kaplan-Meier curve of OS by ethnicity in (**A**) patients undergone surgery compared to those that did not. (**B**) patients that received adjuvant radiotherapy compared those that did not. (**C**) patients that received chemotherapy compared to those that did not. (**D**) patients that received systemic neoadjuvant therapy compared to those that did not

**Fig. 3:**
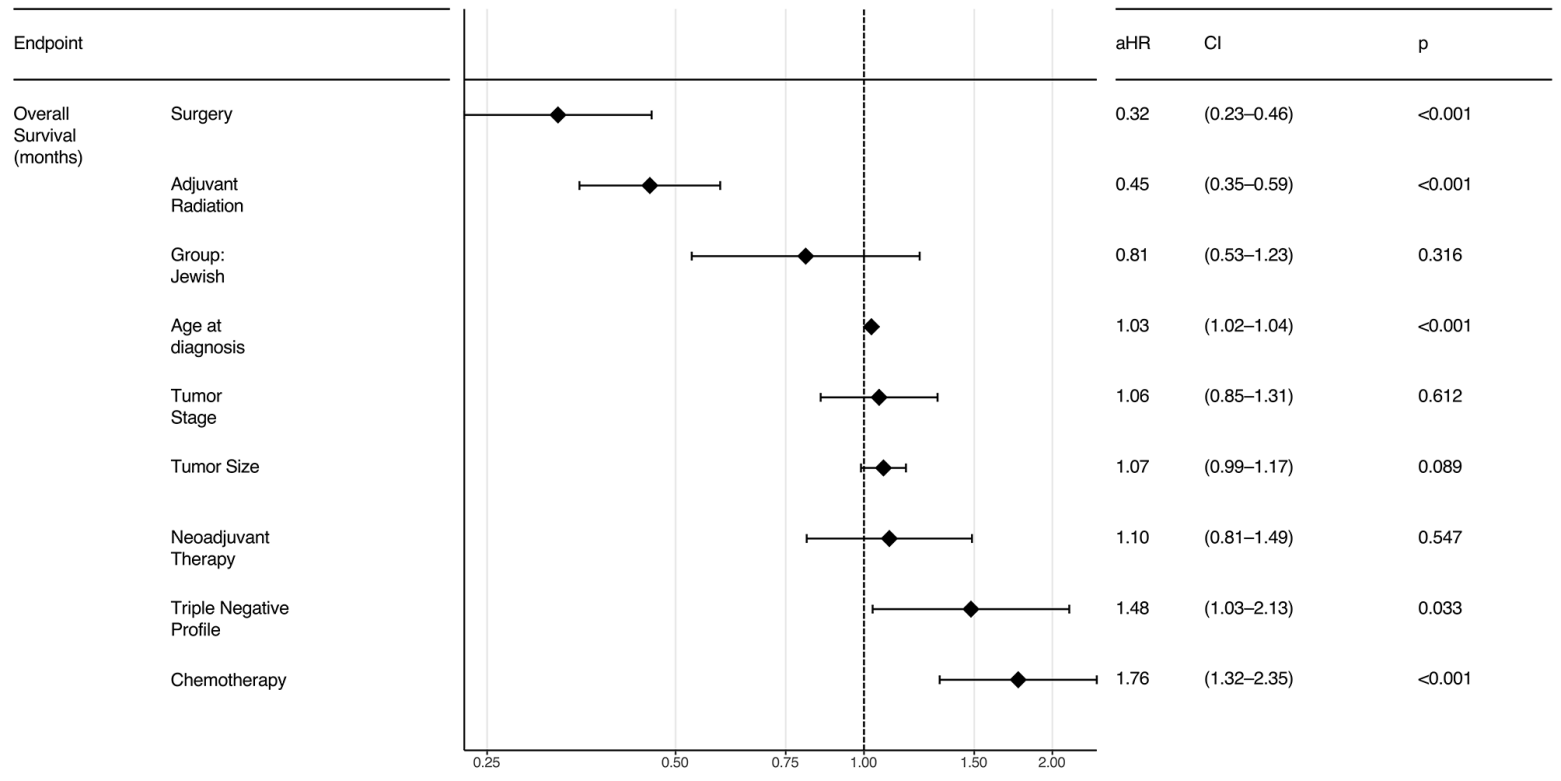
Forest plot showing the association between patient and tumor characteristics and treatment modality with survival in multivariate analysis for early-stage patients. The dashed line represents adjusted Hazard Ratio of 1. HR, hazard ratio. CI, confidence interval

No difference in OS was found between Jewish and Bedouin patients with early-stage disease (127 vs. 126 months, log-rank, *p* = 0.2, Fig. 1A), while Bedouin patients with metastatic disease had better OS (76.6 vs. 37.8months, log-rank, *p* = 0.006, Fig. 1B). No ethnic difference in median OS was observed for the early stage after further stratification (Figures S2, S3, S4). The 5-year and 10-year survival rates for the early-stage disease were 85.3% and 51.2%, respectively, for Bedouin patients, compared to 81.4% and 52.7%, respectively, for Jewish patients. DFS did not differ by ethnicity (not reached vs. 122 months, log-rank, *p* = 0.31, Figure S5). Figure 2 reveals no difference in OS between groups by the following treatment modalities for early-stage patients: surgery (Bedouin vs. Jewish, 126 vs. 135 months, log-rank, *p* = 0.65), adjuvant radiotherapy (126 vs. 128 months, log-rank, *p* = 0.64), adjuvant chemotherapy (126 vs. 110 months, log-rank, *p* = 0.26), and systemic neoadjuvant therapy (126 vs. 113 months, log-rank, *p* = 0.26). Patients with early-stage triple-negative tumors showed no difference in OS by ethnicity (not reached vs. 124, *p* = 0.06, Figure S6). A sub-analysis revealed that Jewish patients with metastatic disease had a significantly higher death rate than Bedouin patients (76.7% vs. 47.2%, *p* < 0.001, Table S1). However, the current study did not differentiate between patients who died from the disease versus those with metastatic disease who died from other causes. OS was similar in Bedouin and Jewish patients less than 70 years old, but in the group older than 70 years, Bedouins had worse OS (Figure S7). The multivariate Cox analysis showed that Jewish ethnicity did not serve as a risk for death, adjusting for demographics, treatment, and tumor features among early-stage patients (adjusted Hazard Ratio 0.81, 95% CI 0.53–1.23, *p* = 0.316, Fig. 3).

## Discussion

In the present study, we examined the epidemiology of breast cancer among Jewish and Bedouin women based on data from SUMC between 2014 and 2021. This study found an interesting pattern of IBC in Bedouin women significantly different from IBC among the Jewish population. While the incidence is higher among the Jewish population, Bedouin women diagnosed with IBC presented at a younger age with larger tumors, a more advanced stage, and more frequently with metastases. A greater incidence of PR was observed in Bedouin women, but there were similar incidences of ER and HER2 IBC subtypes in the two groups of women. No significant differences in OS and DFS were found in patients with early-stage disease; however, in the metastatic group, Bedouin women showed notably longer OS than Jewish women.

This study found a significant difference in the age at diagnosis between Jewish and Bedouin patients. Bedouin women’s mean age at diagnosis was 11 years younger than Jewish patients for early-stage disease and an average of 14 years younger among those with metastatic disease. Previous studies in Israel, Saudi Arabia, the United Arab Emirates, and others have shown a similar young onset, around age 50, among Arab-origin women [18–20]. This finding may be due to differences in disease biology between the two populations, the rapid growth of the younger Bedouin population, and potential under-diagnosis or incomplete registration of IBC in the older population, leading to the under-representation of IBC cases in this group. The Jewish and Bedouin populations are equally entitled to the same health services under the Israeli National Health Insurance Law [21]. Additionally, Israel’s Central Bureau of Statistics has reported no difference in mammography screening adherence between Jewish and Bedouin women since 2011 [22]. Previous studies have highlighted challenges in accessing healthcare for the Bedouin population due to transportation, language, and socio-cultural barriers [23, 24]. However, under-diagnosis or lack of registration is difficult to determine as healthcare accessibility has improved with linguistic adaptation of the healthcare system and increased availability of early cancer diagnosis and treatment in Bedouin settlements and surrounding areas [9, 10].

Our study revealed that Bedouin women are diagnosed at later stages than Jewish women. Although both groups had similar diagnosis rates in Stages 1 and 2 (82% vs. 80%), more Bedouin women were found in Stage 4 (19% vs. 12%). Similarly, Lazarev et al.‘s 2004–2012 data showed more Bedouin women diagnosed at Stage 3 (39% vs. 19%) and Stage 4 (8% vs. 7%) compared to Jewish women [1]. Biologic and minority-associated characteristics might explain such differences [25, 26]. Despite investigations into gene mutations and expression patterns among individuals of Bedouin ancestry from Israel, Qatar, Egypt, the United Arab Emirates, and Kuwait, no genetic markers correlating with the unique breast cancer patterns observed in these populations have been identified, underscoring the need for further research [27–35]. Interestingly, when comparing patients with metastatic disease at presentation, the Bedouin population, though younger, with a more advanced stage at diagnosis and a higher rate of metastasis, had a significantly better OS than the Jewish population. This is possibly due to younger age at diagnosis, which is known to lead to better outcomes [36, 37].

In terms of treatment, modalities such as chemotherapy, neoadjuvant therapy, and adjuvant radiotherapy were more commonly utilized among Bedouin women. This finding may be attributed to their presentation with more advanced stages of non-metastatic disease, highlighting the need for tailored treatments for advanced cancer stages [38]. This observation aligns with the higher incidence of T4 classifications and more advanced nodal involvement within this demographic [1]. Furthermore, when comparing OS and DFS among early-stage patients, no significant differences were found based on ethnicity. However, it’s noteworthy that Lazarev et al. previously reported worse OS and DFS rates among Bedouin women compared to Jewish women [1]. This disparity could be explained by differences in recruitment methods or a too-short median follow-up time, which may not provide substantial OS differences [9, 39–42]. Our study spanned eight years and included Bedouin and Jewish populations. In contrast, Lazarev et al. focused on a narrow timeframe, enrolling 85 Bedouin patients over eight years and 180 Jewish patients within one year. These data collection scope and timing variations could potentially impact differences in OS and DFS rates.

Previous works have shown that early treatment is crucial for improving the survival rate of breast cancer patients [9, 14, 39, 40, 43–46]. Possible differences between Lazarev et al.’s study and the current, in the context of OS and DFS, may be explained by changes in the accessibility of the Bedouin population to tertiary health care centers [2, 9, 10, 39]. However, a governmental effort to enhance healthcare access in the southern region resulted in a 150% increase in clinics within Bedouin settlements from 2008 to 2020, with over 70 clinics serving around 270,000 residents [15]. The introduction and expansion of mobile mammography units in 2006 and 2011 might have also improved breast cancer screening accessibility [14]. Additionally, the SUMC cancer center opening in 2018, which upgraded both inpatient and outpatient oncology facilities, likely further advanced care, and screening opportunities for Bedouin women [9, 42]. These expanded healthcare resources might have alleviated the disparities by facilitating earlier diagnosis, treatment, and post-treatment monitoring, potentially influencing the observed enhancements in OS and DFS.

While studying the epidemiology of breast cancer in Jewish and Bedouin women, we should consider the significant societal changes the Bedouin community is undergoing. Traditionally, Bedouin society has been a semi-tribal, isolated minority emphasizing family, community, and cultural and traditional beliefs [11, 12, 16, 47]. However, in recent times, Bedouin society has become more modern and urbanized, driven by improved access to education and employment, modern technology, media exposure, and urbanization [11].

### Strengths and limitations

Our single-site retrospective cohort study, while limited by its timeframe for patient selection, benefits from a large sample size and comparable screening characteristics across populations. Limitations in the data gathered on breast cancer known factors such as method of diagnosis (symptoms or mammography), parity, BMI, diabetes, and family history of IBC, prevented us from comparing the prevalence of these factors and may have led to a confounding bias. Moreover, the lack of data regarding the Ki-67 marker precluded the ability to analyze IBC subtypes.

## Conclusion

Breast cancer in Bedouin women in southern Israel is characterized by younger age at diagnosis, larger tumors, and more advanced stages compared to Jewish women. Despite historical disparities in prognosis for breast cancer, current data show no ethnic differences in OS and DFS. Enhanced research, including a more extensive database and molecular cancer markers analysis, is crucial for understanding the mechanisms for the disparities between Bedouin and Jewish women.

### Electronic supplementary material

Below is the link to the electronic supplementary material.

Supplementary Material 1

Supplementary Material 2

Supplementary Material 3

Supplementary Material 4

Supplementary Material 5

Supplementary Material 6

Supplementary Material 7

Supplementary Material 8

## Data Availability

The datasets generated and analyzed during the current study are not publicly available because we intend to use the database for future studies and analysis. However, the datasets are available from the corresponding author on reasonable request.
