# Supplementary material for: Epidemiological, clinical, and pathological characteristics of invasive breast cancer in Bedouin and Jewish women in southern Israel: a retrospective comparative study
Source: BMC Cancer. 2024 Mar 6;24:310. doi: 10.1186/s12885-024-12051-w (PMC10916252; doi:10.1186/s12885-024-12051-w)
Supplement: Supplementary file 1 — Supplementary Material 1 [file 12885_2024_12051_MOESM1_ESM.docx]

| Patient and  Tumor Characteristics | All  N = 248 | Jewish  N = 193 | Bedouin  N = 36 | p value |
| --- | --- | --- | --- | --- |
| Age, years, mean (SD) | 59 (16.4) | 61 (16.2) | 47 (12.5) | **< 0.001** |
| Tumor size, cm (SD) | 3 (1.89) | 3 (1.9) | 4 (1.8) | 0.077 |
| ER positive, n (%) | 169 | 146 (79.3%) | 23 (65.7%) | 0.084 |
| PR positive, n (%) | 139 | 116 (63%) | 23 (65.7%) | 0.849 |
| HER 2 positive, n (%) | 71 | 57 (31) | 14 (40%) | 0.327 |
| Triple Negative | 24 | 21 (11.4%) | 3 (8.6%) | 0.774 |
| Death | 165 | 148 (76.7%) | 17 (47.2%) | **< 0.001** |

**Table S1**

**Table S1.** Baseline demographics and tumor characteristics for patients with metastatic disease. SD, standard deviation; ER, estrogen receptor; PR, progesterone receptor; Her 2, human epidermal growth factor receptor 2.

**Figure S1.** Boxplot (A) of age at diagnosis in Bedouin and Jewish patients. Boxplot (B) of tumor size in cm between the two groups. Outliers are shown as solid circles.


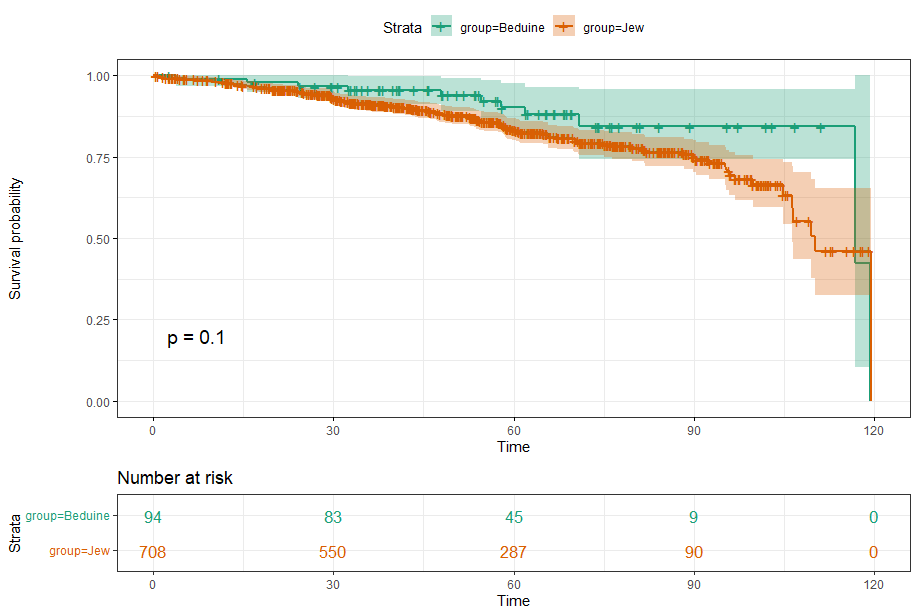
 **Figure S2.** Stage 1 OS by ethnicity


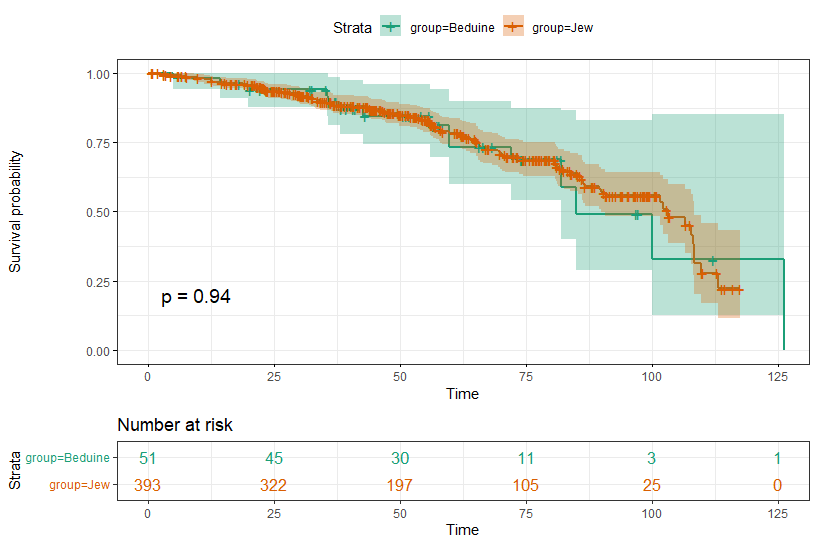
 **Figure S3.** Stage 2 OS by ethnicity

**Figure S4.** Stage 3 OS by ethnicity

**Figure S5.** DFS by ethnicity

**Figure S6.** Triple negative OS by ethnicity ****

**Figure S7**. Kaplan-Meier curve of OS in Bedouin and Jewish patients divided by age group: (A) less than 50 years old, (B) between 50 to 70 years old, and (C) more than 70 years old.
